# Magnetic Compass Orientation in the European Eel

**DOI:** 10.1371/journal.pone.0059212

**Published:** 2013-03-15

**Authors:** Caroline M. F. Durif, Howard I. Browman, John B. Phillips, Anne Berit Skiftesvik, L. Asbjørn Vøllestad, Hans H. Stockhausen

**Affiliations:** 1 Institute of Marine Research, Austevoll Research Station, Storebø, Norway; 2 Department of Biological Sciences, Virginia Polytechnic Institute and State University, Blacksburg, Virginia, United States of America; 3 Department of Bioscience, Center for Ecological and Evolutionary Synthesis, University of Oslo, Oslo, Norway; 4 Institute of Marine Research, Bergen, Norway; University of Lethbridge, Canada

## Abstract

European eel migrate from freshwater or coastal habitats throughout Europe to their spawning grounds in the Sargasso Sea. However, their route (∼ 6000 km) and orientation mechanisms are unknown. Several attempts have been made to prove the existence of magnetoreception in *Anguilla sp.*, but none of these studies have demonstrated magnetic compass orientation in earth-strength magnetic field intensities. We tested eels in four altered magnetic field conditions where magnetic North was set at geographic North, South, East, or West. Eels oriented in a manner that was related to the tank in which they were housed before the test. At lower temperature (under 12°C), their orientation relative to magnetic North corresponded to the direction of their displacement from the holding tank. At higher temperatures (12–17°C), eels showed bimodal orientation along an axis perpendicular to the axis of their displacement. These temperature-related shifts in orientation may be linked to the changes in behavior that occur between the warm season (during which eels are foraging) and the colder fall and winter (during which eels undertake their migrations). These observations support the conclusion that 1. eels have a magnetic compass, and 2. they use this sense to orient in a direction that they have registered moments before they are displaced. The adaptive advantage of having a magnetic compass and learning the direction in which they have been displaced becomes clear when set in the context of the eel’s seaward migration. For example, if their migration is halted or blocked, as it is the case when environmental conditions become unfavorable or when they encounter a barrier, eels would be able to resume their movements along their old bearing when conditions become favorable again or when they pass by the barrier.

## Introduction

European eels (*Anguilla anguilla*) undertake long-distance migrations between their spawning grounds in the Sargasso Sea and their inland and coastal habitats in Europe and North-Africa [1], [2]. Small larvae drift with the Gulf Stream to reach their destinations in Europe. After active upstream migration, they settle in extremely diverse habitats ranging from brackish water marshes and marine coastal areas to freshwater rivers and lakes, sometimes up to thousands of kilometers upstream. When the fish reach sexual maturity, up to 20 years after their arrival, they migrate down river systems, navigate coastal areas and then swim across the Atlantic Ocean to their spawning grounds. Eels form a panmictic population [3]. There is no known geographic or temporal genetic segregation for this species. This has been interpreted to mean that eels from all over Europe meet their conspecifics at a common spawning location which has yet to be found.

Eels also display seasonal migrations within a river system and between fresh- and saltwater habitats [4]. They change their territories during transitional periods between summer and winter. Temperature is a driver for these migrations as eels avoid cold waters [5], [6], [7]. Movements are directed to warmer waters or places where they can burrow in sand and mud to overwinter [2]. Habitat transitions usually occur at temperatures around 12°C, below which eels decrease their activity [8], [9], [10].

Although temperature can function as an imprecise orientation cue, eels require an orientation/navigation system as a guidepost to orient since no coastline or bottom structure is available during their journey across the Atlantic Ocean. As for temperature, salinity and odor are unlikely orientation cues because the gradients in these variables over thousands of kilometers are inconsistent and small. It is also unlikely that optical features of the sky (sun, stars, polarization) are used by eel, since they migrate mainly at night and often travel at great depth [11]. The Earth’s magnetic field can provide the necessary cues - compass orientation and navigation - needed to travel long distances in an environment with few or no alternate guideposts [12].

Both behavioral and electrophysiological responses to magnetic fields have been observed in fishes. Sockeye salmon (*Oncorhynchus nerka*) alevins and smolts changed their directional preference with shifts in the horizontal component of the magnetic field [13], [14]. Conditioning experiments showed that yellowfin tuna (*Thunnus albacores*) could discriminate between Earth-strength magnetic fields of different intensities and inclinations [15]. Rainbow trout (*Oncorhynchus mykiss*) learn to discriminate between the presence and absence of a magnetic anomaly and are sensitive to inclination, intensity and direction of the magnetic field [16], [17], [18]. Neural responses to changes in the direction and the intensity of the magnetic field have been recorded from the trigeminal system of this fish [16]. A magnetic sense has also been observed in non-migratory fishes. Significant bimodal orientation and alignment was found in zebrafish and carp [19], [20], [21], but no evidence for a magnetic sense was found in goldfish [22].

Because of its lengthy migration, *Anguilla sp*. was among the first animals to be tested for magnetic orientation [23]. However, earlier studies failed to show consistent orientation relative to the magnetic field [24], [25], [26], presumably because they were carried out in non-uniform magnetic fields [27], [28], in the presence of large electrical artifacts [29], [30], [31], or at magnetic intensities that were orders of magnitude above that of the Earth’s magnetic field (e.g. [32]).

The objectives of this study were to determine 1. whether conditions could be identified in the laboratory that would elicit consistent orientation by European eels relative to an earth-strength magnetic field, and 2. whether European eel can orient relative to an earth-strength magnetic field under controlled laboratory conditions.

## Materials and Methods

### Ethics Statement

No permits were required by the Norwegian authorities for collection of eels or to carry out these experiments since no eels were harmed in this study.

### Test Fish

**Figure 1:**
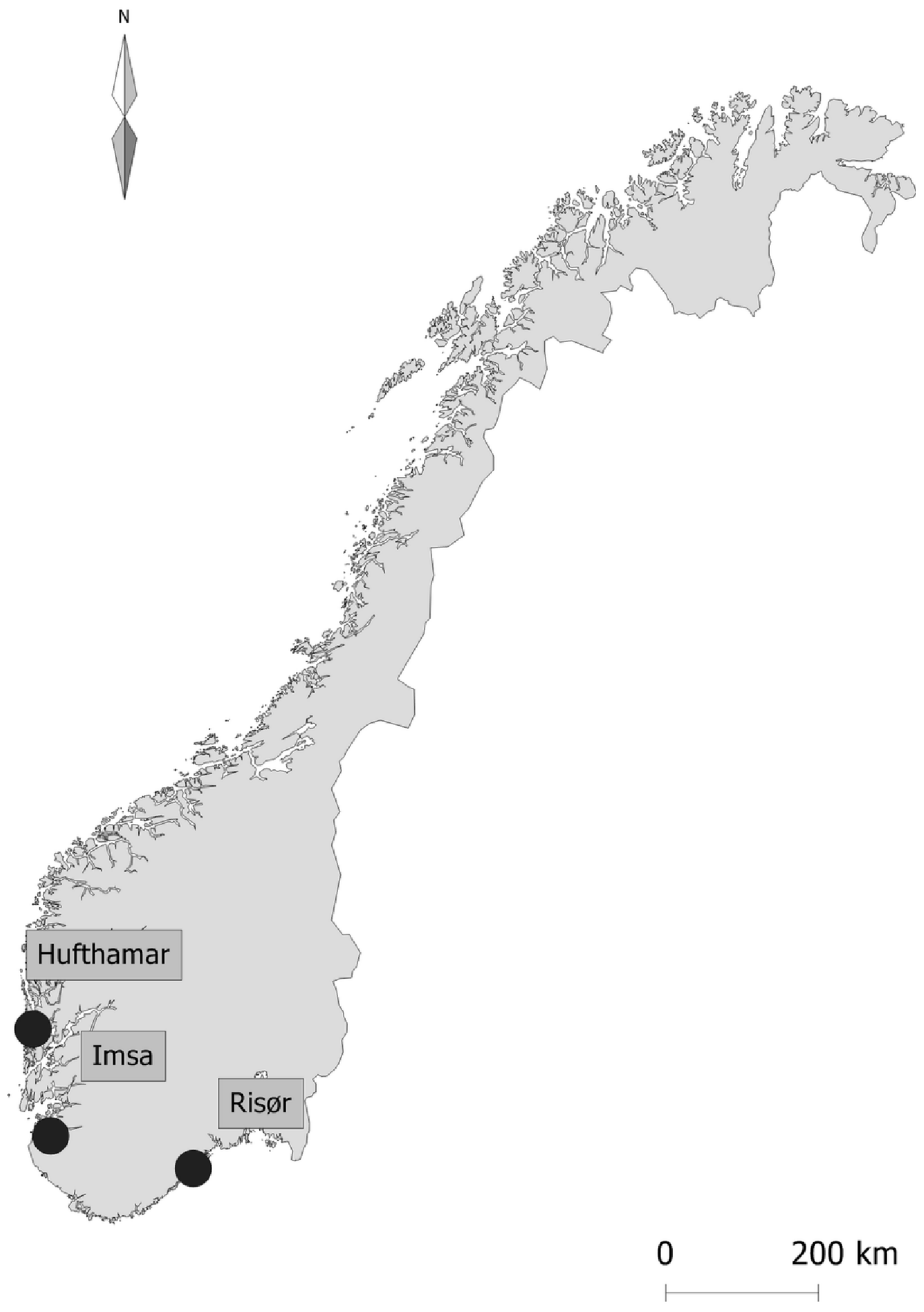
Location of sampling sites of eels and testing facility.

The European eels (hereafter “eels”) tested in these experiments were collected at two locations (Fig. 1): the river Imsa (58.9 N and 5.9 E) in western Norway and along the Skagerrak coast (58.72 N and 9.22 E) in southern Norway. Imsa eels were caught in a trap (NINA aquatic research station) as they were leaving the river presumably on their reproductive migration. Skagerrak eels were caught using eel pots by commercial fishers. This particular fishing gear targets resident eels and, therefore, most of these eels were at the yellow stage, but some individuals showed signs of silvering. The stage of eels was determined according to Durif et al. [33]. Eels were transported by car in oxygenated water to the Institute of Marine Research’s (IMR) research station on the archipelago of Austevoll, Norway (60.09 N and 5.26 E).

### Testing and Training Tanks

**Figure 2:**
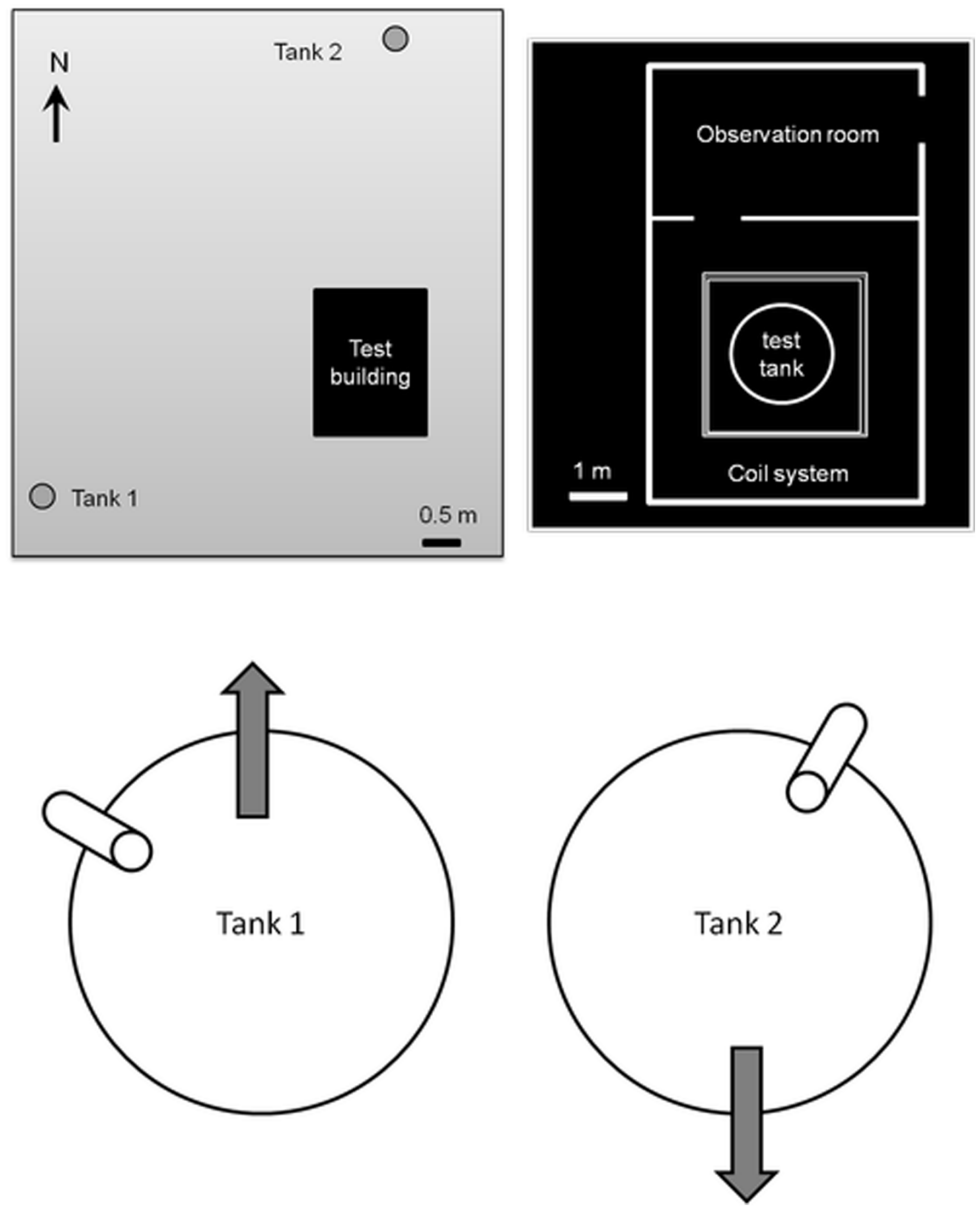
Schematic drawing of the test building and training tanks. Distances are to scale (the scale is indicated above the black line). Circles indicate the position of the training tanks. Details of the training tanks show the location of the water inflow (cylinders) and the directions eels were taken out of the training tanks.

**Figure 3:**
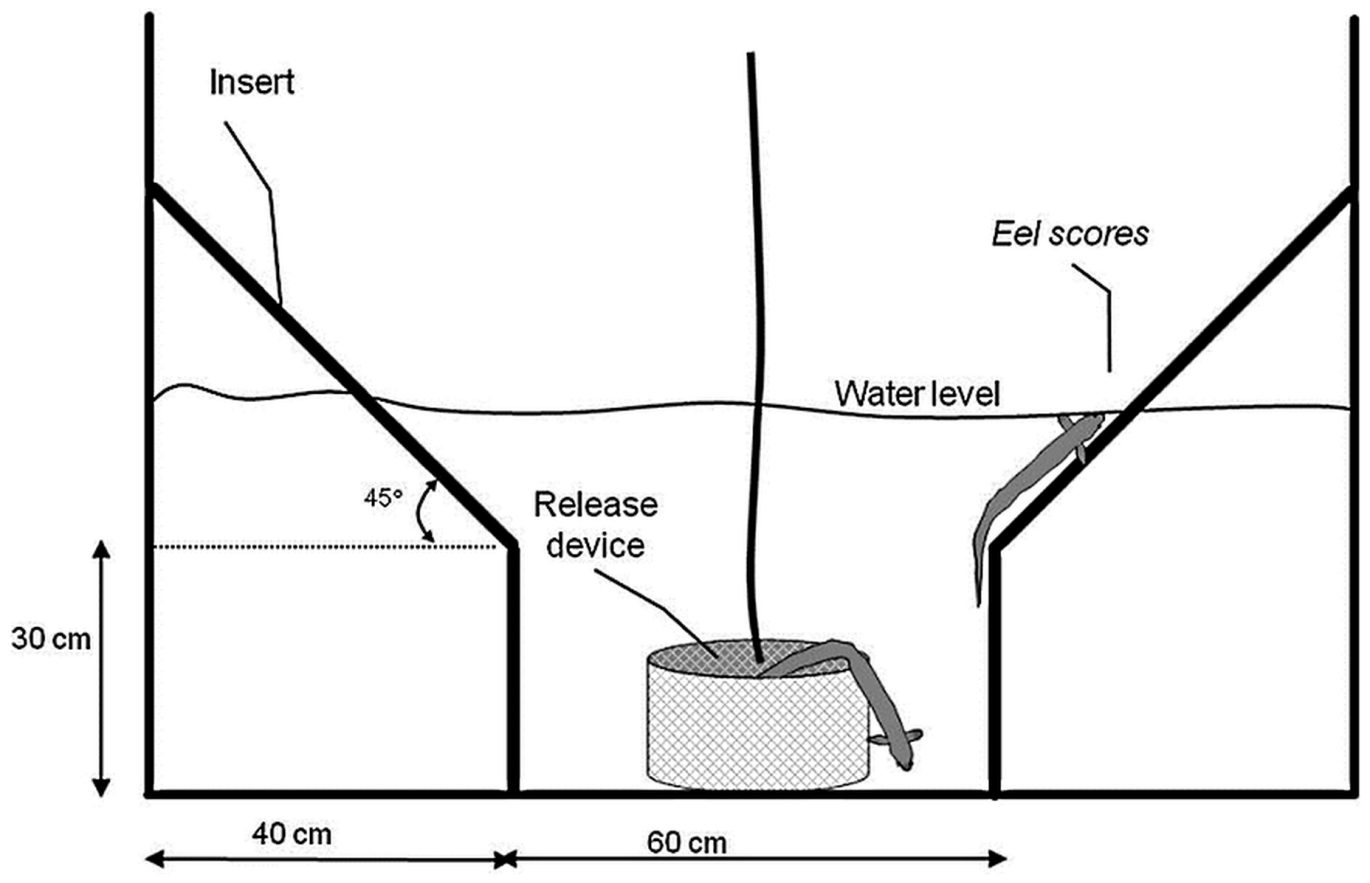
Schematic of the test tank and funnel insert. Once the release device is lowered into the tank, the eel is able to come out in any direction. Its escape direction (where it touches the water surface along the slopes of the funnel) is recorded as a bearing.

Testing was conducted at IMR’s magnetic orientation facility (60.12 N and 5.21 E: Hufthamar, Austevoll, Norway), 9 km northwest of the research station. At this latitude, declination is less than 1° W. The test building (built out of non-magnetic material) is located in a field around 145 m away from the nearest electrical disturbance (power generator, high power cables, and buildings). To ensure that the site was not subject to any magnetic anomaly, the area around the building was mapped using a Geometrics 816/826A proton precession magnetometer (H.H. Stockhausen, unpublished). The building houses the test tank, the coil-system as well as the electrical and video recording equipment. Saltwater is pumped directly from the sea (400 m away) into a header tank that supplies two outside training tanks and the test tank (Fig. 2). The test tank sits on a pedestal so that the bottom part of the tank coincides with the middle of the coil system where the magnetic field is the most homogeneous. The pedestal and test tank sit on an independent concrete plate so that walking around the test tank does not cause any vibrations in the water. The test tank measures 1.40 m in diameter and 0.90 m in height. It is fitted with a black hexagonal funnel-like PVC insert (Fig. 3). The inner vertical part of the funnel measures 30 cm and is 60 cm wide. It then slopes out on the sides. During each test, the behavior of one animal was recorded in complete darkness using an infrared camera located above the test tank.

At least two days before testing, eels were divided into two groups and moved to the testing facility. A group of eels was placed in one of the two training tanks (diameter = 1.20 m, height = 1 m). The only cues that differed between the training tanks, other than those associated with the location, were the directions of water inflow. In training tank 1, the continuous inflow of seawater was supplied from a pipe located at 30° relative to magnetic north. In training tank 2, the inflow was located at 300°. Tanks were covered with a black PVC lid. Water was drained from a pipe in the center of the tank.

The training tanks were located 25 m away from the test building but on opposite sides of the building (Fig. 2). Pipes (approximately 60 cm in length) were placed inside the training tanks for shelter. These floated at the water surface and their alignment changed irregularly as a consequence of the water current coming from the inflow.

Seawater in the test tank and in the training tanks came from the same header tank but the water temperature inside the building was always 1–2°C higher than in the training tanks when the tests started.

### Magnetic Coil System

Electricity was provided by a generator located 220 m away. Electric current was routed through an uninterruptible power supply (UPS) to stabilize it. The UPS was connected to an adjustable multichannel power supply and then to the switchbox that controlled the coil system.

The cube coil system follows the design of [34], (see also [35]) with a set of four double-wrapped coils. One coil was used to cancel the horizontal component of the ambient field and the remaining three coils were used to produce artificial magnetic fields matching the intensity and inclination of the ambient field and aligned in one of four directions with magnetic north at geographic north, east, south, or west. Bearings were pooled from an approximately equal number of eels tested in each of the four magnetic field alignments (each eel was tested only once). This made it possible to factor out any consistent non-magnetic bias and retain only the component of orientation that was a response to the magnetic field.

During each test, the eel remained in an area in the center of the coil system restricted by the funnel-like insert which corresponded approximately to a cylinder (30 cm radius, 35 cm length) inside of which the magnetic field was uniform [35]. Magnetic field values were recorded using a three axis Applied Physics 520 fluxgate magnetometer during each test. Total intensity inside the coil system was set to replicate as closely as possible total intensity of the ambient field and varied from 50.3 µT to 51 µT. The deviation from the inclination of the ambient field (73°) was <1°.

### Testing Protocol

One of the main difficulties in establishing a protocol to test responses by animals to magnetic fields resides in eliciting an observable response; in our case, finding a criterion that will reveal the eel’s directional preference. Previous studies on eels used body position, success in traversing mazes, or escape behavior along the tank walls. However, eel behavior is unpredictable and under natural conditions many days can pass before they move. Even at the migratory (silver) stage, eels can remain motionless for several days [36], [37].

In our study, we carried out experiments under different temperature conditions spanning the threshold for triggering eel migration, *i.e*.: 6–17°C. Trials were carried out between April and October 2010 during daytime. Each individual was tested once, in one of the four alignments of the magnetic field in the testing tank. Subjects were tested alternately from the two holding tanks. For each trial, the artificial magnetic north direction was preset inside the testing tank at geographic north, south, east, or west.

Eels were displaced from two outdoor holding tanks, located in opposite directions from the indoor testing tank. The observer collected an eel from one of the training tanks by removing one of the floating shelter pipes, transporting the eel into the testing building in the pipe, and allowing the eel to slide out of the pipe into the release device, which consisted of an open plastic basket. The eel was always taken out of the water on the same side of the training tank (tank 1: c. 360°; tank 2: c. 180°). The paths taken from the training tanks to the test building were not straight and the transfer tube was swung from side to side during the displacement to the test building (over approximately 20 m). Once the eel was transferred from the tube to the release device inside the test building (lights off), the release device was hooked to a pulley above the center of the test tank, and again spun so that it revolved around its vertical axis to disorient the eel while the observer quietly left the room. The observer then lowered the release device from the observation room into the water to release the eel. There was no water flow in the testing tank while eels were being tested.

After release, the animal’s behavior was recorded using an IR camera under IR LED illumination over an 11-minute sequence. This time period was chosen based on preliminary experiments on the behavior of eels in the testing tank (Durif, unpublished). Typically, the eel would first swim out of the immersed release device and settle to the bottom of the tank. It then circled along the bottom and then made vertical incursions, finally choosing a direction along the sloped panels (Fig. 3). The location where the eel swam up the sloped side of the funnel and contacted the water surface were recorded as an escape direction (ED). After ∼10 minutes, the eel would generally stop swimming and remain stationary at the bottom of the tank. Approximately 6 trials were conducted in one day. As expected, eels showed no or very little movement at temperatures <6°C and those tests yielded no results. After each trial, the insert was scrubbed with a mop to remove or spread any olfactory cue left by the previously tested individual. The water was changed from day to day but not between trials conducted on any one day.

### Data Analysis

Equal numbers of eels were tested in the four horizontal magnetic fields (north, south, east, and west) to factor out any consistent topographical bias. However, some eels never moved resulting in a slightly unequal design (tests in the north field: 14; south field: 13 east field: 14, west field: 12 tests). Videos were later analyzed by a blind observer with no knowledge of the artificial direction of magnetic North that was set. The Rayleigh test was used to determine if the distributions of the mean bearings of individual eels were non-randomly distributed [38]. Statistics for bimodal distributions were calculated by doubling each data value and then testing using the Rayleigh test. The V test was used to assess whether the observed angles had a tendency to cluster around the direction of displacement [38]. The Watson’s U test was used to compare the mean distribution of yellow and silver stage eels [38]. The circular-linear correlation (*r*’) between temperature and orientation data was calculated according to [39].

## Results

The escape directions (ED) of eels were recalculated relative to the direction of the artificial magnetic north in the testing tank. For example, if ED was 35° and the alignment of magnetic north during testing was to the west (270°), the “magnetic bearing” would be 125°. Bearings were also standardized according to the direction of their displacement away from the holding tank (tank 1: c. 360°; tank 2: c. 180°).

**Figure 4:**
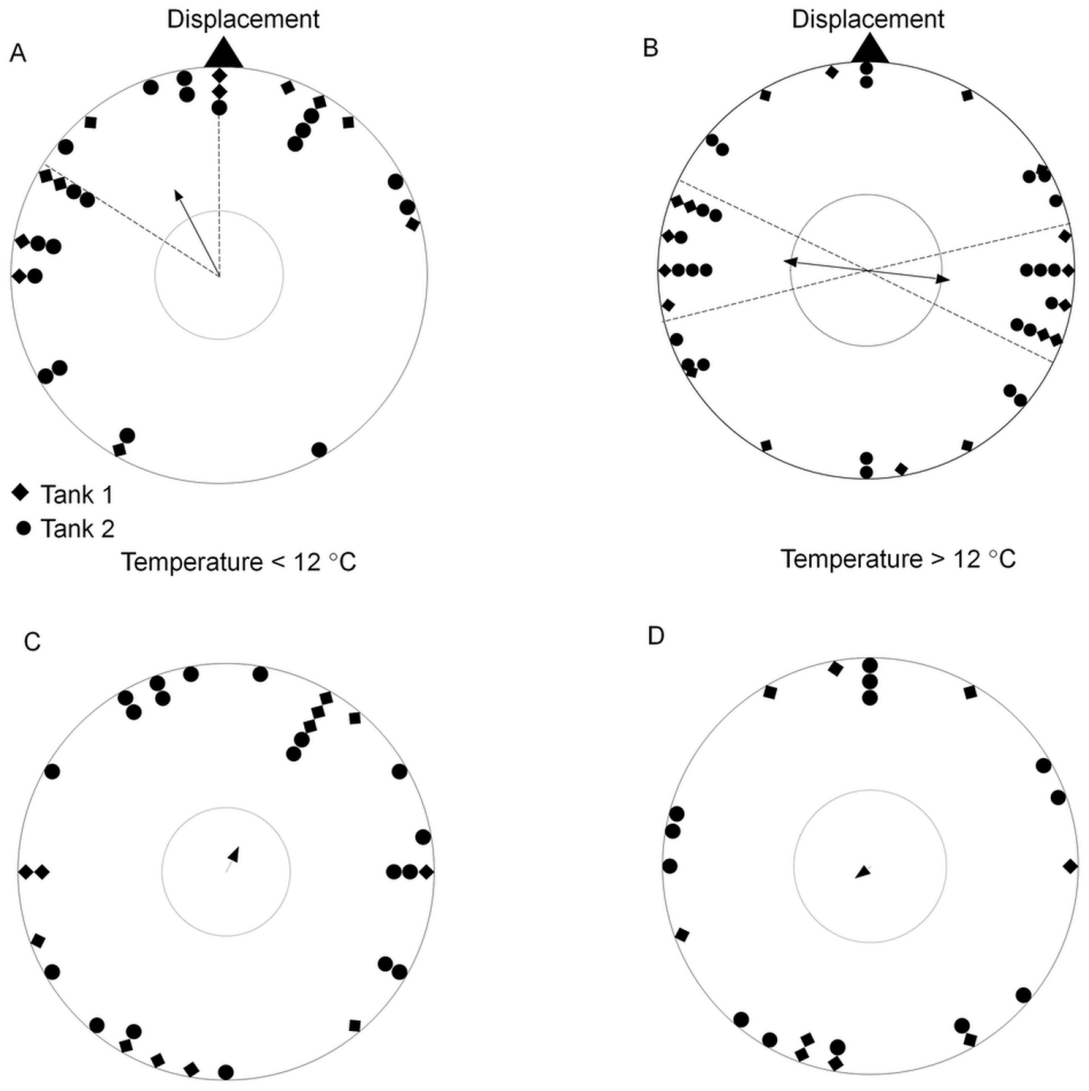
Orientation of *Anguilla anguilla* under four artificial magnetic conditions at temperatures between 6 and 17 °**C.** Eels taken from training tank 1 are represented by diamonds and eels taken from training tank 2 are denoted by circles. Bearings (relative to magnetic North) were standardized relative to the direction of displacement. The triangular symbol represents the direction of displacement. The center arrow shows the mean angle of the group weighed by r (scaled 0–1) and the 95% confidence interval. The inner circle represents a significance level of 5% for the Rayleigh test. A and B are bearings standardized to the direction of the magnetic field; C and D are topographical bearings (relative to geographic north). A and C: Tests carried out at temperatures <12°. B and D: Tests carried out at temperatures >12°. Bearings on B have been doubled as they displayed significant bimodal distribution.

Magnetic bearings were significantly correlated with water temperature in the training tanks (6°<temperature <17°; circular-linear correlation: *r’* = 0.45, *n* = 53, *p*<0.001). At temperatures below 12°C, eels significantly oriented in the direction of displacement (Fig. 4A; *µ* = 331°, *r* = 0.46, *n* = 31, *p = *0.001). At temperatures above 12°C, the distribution of magnetic bearings was indistinguishable from random (*r* = 0.25, *n* = 22, *p = *0.25); however magnetic bearings displayed bimodal distribution along an axis of 97–277° (Fig. 4B; *r* = 0.40, *n* = 22, *p = *0.027). The two distributions (low and high temperature) were significantly different (*U^2^* = 0.683, *df_1_* = 22, *df_2_* = 31, *p*<0.001). The V tests showed that at low temperature, bearings were significantly clustered around the direction of displacement (expected mean: 360°, *V* = 0.40, *p*<0.001) but not at high temperature at which bearings were perpendicular to displacement (Fig. 4B; expected mean: 360°, *V* = −0.046, *p* = 0.62).

The distribution of magnetic bearings did not differ significantly between yellow and silver stage eels (*U^2^* = 0.06, *df_1_* = 22, *df_2_* = 31, *p*>0.5).

The distribution of EDs relative to geographic north (*i.e*. ignoring the alignment of the magnetic field in the testing) was random both below and above 12°C (Fig. 4C and 4D; respectively: *p* = 0.56 and *p* = 0.83). This shows that there was no consistent non-magnetic orientation in the testing arena due to topographical cues (any visual, olfactory or auditory asymmetries in the testing tank).

## Discussion

Eels exhibited a consistent direction of orientation relative to the magnetic field. The directional preference was specific to the location of the training tank in which they were held prior to testing. The directional preference corresponded to the direction of displacement away from the training tank and not to any other cue (such as water inflow, for example). An analogous response was observed in earlier studies of newts, in which individuals continued to orient in a direction away from the training tank when they were displaced along the shoreward axis away from their training tank [40], [41], [42]. In our study, the water in the test tank was 1–2°C higher than in the training tank. Thus, the displacement towards the test tank resulted in a more favorable water temperature, perhaps reinforcing the eel’s preference for the displacement direction. As expected, the directional response was stronger at lower testing tank temperatures (6–11°C) compared to higher temperatures (12–17°C), although in both cases the water temperature in the testing tank was 1–2°C higher than in the training tanks. The lower temperature range in the testing tank corresponds to the “environmental window” in which eels migrate [7], [43]. Eels avoid cold water [5] at all life history stages. Hence, in our experiment, yellow and silver eels did not behave differently: mean escape directions were not significantly different for the two life stages.

Registering the direction of passive displacement and subsequently orienting in the same direction may also be a way for eels to regain the main flow of a river system when they encounter local turbulence and possibly contradictory cues. Eel frequently stop during their downstream migration when environmental conditions are not favorable (*i.e*. periods of low water flow, as well as during daylight hours or when turbidity is low, presumably to avoid detection by predators). Telemetry studies demonstrate that eels typically rest in places where there is no current [36]. In the absence of current flow, using compass cues to reorient in their previous direction of displacement (*i.e.* the river axis) would allow them to resume their migration when environmental conditions become favorable.

This behavior would also be useful during their oceanic migration where no coastline or bottom structure is available to provide them with cues for orientation. Eels display diel vertical migrations of amplitudes of up to 800 m between day and night [11]. They may rest on the seabed during daylight [44]. Flow direction often changes vertically in the water column, and flow rate is low (almost zero) near the seafloor. Remembering the magnetic compass direction of their previous migratory path would have an obvious advantage in an environment devoid of any topographical cues.

At higher temperatures (12–17°C), eels exhibited bimodal orientation along an axis perpendicular to the axis along which they were displaced. If displacement corresponded to downstream movements along the river axis, then bimodal orientation would represent y-axis orientation, defined by Ferguson and Landreth [45] as movement perpendicular to a shoreline towards either land or deep water. The use of a magnetic compass for y-axis orientation has been demonstrated in amphibians, freshwater fish, and turtles [40, 46, 47, 48 and references therein]. In eels, orientation along the y-axis at higher temperature would occur during spring and summer: eels will search for food in the more shallow areas along the banks while finding refuge from predators and high temperature in deeper water. Hence, a bimodal orientation perpendicular to the axis of displacement - which eels demonstrated in our experiments - is consistent with their behavioral ecology at higher temperature.

Using a protocol that eliminated topographical cues such as olfactory (odor trails in the water), mechanical (vibrations caused by the observer), visual or auditory cues in the test building, we showed that eels are able to orient relative to the magnetic field. Eels tagged and released at sea can maintain a compass direction [44], [49]. Here, we present evidence indicating that they are likely to use the Earth’s magnetic field to do so. Eels in the present experiments did not orient according to an innate course, as the orientation directions of the two groups were clearly opposite and linked to the training tank. Therefore, it appears that they are able to register a direction which they can subsequently use to guide their movements.

Whether eels can sense large scale gradients in the inclination or the intensity of the magnetic field to determine geographic position still has to be tested by simulated magnetic displacements (*e.g.* [50]). Future experiments at the Austevoll magnetic orientation facility, involving different values of magnetic inclination and intensity simulating a displacement to the Sargasso Sea area, may provide new insights into the location of the European eel’s spawning grounds.
